# Metformin promotes osteogenic differentiation and prevents hyperglycaemia-induced osteoporosis by suppressing PPARγ expression: Metformin prevents osteoporosis by suppressing PPARγ

**DOI:** 10.3724/abbs.2023043

**Published:** 2023-03-23

**Authors:** Lifeng Zheng, Ximei Shen, Yun Xie, Hong Lian, Sunjie Yan, Shizhong Wang

**Affiliations:** 1 The First Affiliated Hospital of Fujian Medical University Fuzhou 350005 China; 2 Orthopedics Department the First Affiliated Hospital of Fujian Medical University Fuzhou 350005 China; 3 Endocrinology Department the First Affiliated Hospital of Fujian Medical University Fuzhou 350005 China; 4 Diabetes Research Institute of Fujian Province Fuzhou 350005 China; 5 Fujian Medical University Fuzhou 350005 China

**Keywords:** metformin, osteoporosis, diabetes, PPARγ, endoplasmic reticulum stress

## Abstract

Metformin can prevent hyperglycaemia-induced osteoporosis and decrease the bone fracture rate, but the mechanism has not been fully elucidated. To reveal the mechanism by which metformin affects hyperglycaemia-induced osteoporosis, we treat a mouse osteoporosis cell model with metformin and find that osteoblast mineralization increases and PPARγ expression decreases. Single-cell mRNA sequencing analysis show that PPARγ is highly expressed in the bone tissue of osteoporosis patients, which highlights the role of PPARγ in osteoporosis. Furthermore, we find that PPARγ is the effector through which metformin prevents osteoporosis. We further examine the mechanism of PPARγ regulation and reveal that metformin regulates PPARγ expression through the AMPK pathway and that PPARγ affects osteoblasts through the endoplasmic reticulum stress (ERS) pathway. Moreover, we verify the association between the effect of metformin on bone metabolism and the expression of PPARγ in high-fat diet-induced diabetic rats. Thus, we identify and functionally validate that metformin prevents hyperglycaemia-induced osteoporosis by regulating the AMPK-PPARγ-ERS axis.

## Introduction

The number of people with diabetes mellitus (DM) has quadrupled in the past three decades worldwide and may grow to 693 million by 2045 [ 1, 2] . The prevalence of osteoporosis was reported to be 23.1% in women and 11.7% in men worldwide [3]. However, the prevalence rate of osteoporosis in type 2 diabetes mellitus (T2DM) patients is 32.1% in women and 21.2% in men [4]. In addition, bone fragility is a typical complication of diabetes, and the risk of fragility fractures is increased in diabetes patients [5]. Thus, diabetes is a risk factor for osteoporosis and fragility fractures.

Metformin is a widely used medication for the management of diabetes and related areas of clinical treatment and appears to function via multiple pathways [ 6– 8] . Multinomial clinical studies have shown that metformin increases bone mineralization, reducing fracture risk in diabetes patients [9]. Cell line experiments also revealed that metformin improves bone formation by osteoblasts [10]. However, the molecular mechanism by which metformin protects osteoblasts has not been fully elucidated.

Peroxisome proliferator-activated receptor γ (PPARγ) is a ligand-inducible transcription factor that is a member of the nuclear receptor superfamily and plays a key role in the regulation of cell differentiation and lipid metabolism [11]. Adenosine 5′-monophosphate-activated protein kinase (AMPK) activation and an increase in BMP2 are negatively correlated with the expression of PPARγ [ 12, 13] . PPARγ correlates with insulin resistance and is considered to be a drug target for treating T2DM [14]. In addition, PPARγ activation can affect the structure of osteoblasts [15] and result in the loss of bone mass in the context of diabetes [16]. Thus, targeting PPARγ may be a promising approach to prevent osteoporosis. However, the regulation of PPARγ activity and the downstream effects of PPARγ on hyperglycaemia-induced osteoporosis are still unclear.

Hyperglycaemia can lead to metabolic disturbances via multiple pathways, such as the activation of Toll-like receptors (TLRs), inflammasome activation and endoplasmic reticulum stress (ERS) [17]. TLRs regulate osteoclast genesis and bone resorption through myeloid differentiation and β-interferon pathways [18]. The NLRP3 inflammasome plays a key role in the pathogenesis of osteoporosis by affecting the differentiation of osteoblasts and osteoclasts [19]. ERS induces osteoblast apoptosis and is related to metabolic bone disease [20]. In β-cells, hyperglycaemia-induced ERS is inhibited by metformin treatment [21] and PPARγ activation [22]. In human bronchial epithelial cells, AMPK alleviates ERS by inducing the ER chaperone ORP150 [23], and AMPK is closely related to the expression of PPARγ. However, the interplay among metformin, ERS and PPARγ in osteoblasts is still unknown.

In this study, to examine the regulatory relationship between metformin and the AMPK-PPARγ-ERS axis in osteoporosis prevention, we treated mouse osteoblasts and a diabetic rat model with metformin and then explored the mechanism by which metformin affects hyperglycaemia-induced osteoporosis.

## Materials and Methods

### Cell culture

Rats were used for the animal model, and the cell line model was mouse derived. Cell culture was used as previously described [24]. Briefly, the MC3T3-E1 subclone 14 cell line (BDCB, Guangzhou, China) was cultured in base medium [Eagle’s medium supplemented with 5.6 mM glucose (Biofroxx, Einhausen, Germany), 2 mM L-glutamine (Solarbio, Beijing, China), 0.5 mM β-glycerophosphate (Beyotime, Shanghai, China), 50 mg/L ascorbic acid (Beyotime), and 10% fetal bovine serum (PAN Biotech, Aidenbach, Germany)] with 5% CO _2_ at 37°C. The (seeding) cell density was 3×10 ^5^ cells/mL, and cells at passages 15 to 18 were collected. Before processing, the differentiation capacity of MC3T3-E1 cells was checked at passages 15 to 18.

For high glucose intervention, MC3T3-E1 cells were cultured in high glucose medium (base medium supplemented with 25 mM glucose) for 14 days. For metformin intervention, MC3T3-E1 cells were cultured in medium containing different concentrations of metformin (25, 50, 100 μM metformin in base medium) for 14 days. For thapsigargin intervention, MC3T3-E1 cells were cultured in base medium containing 100 nM β-thapsigargin (Beyotime) for 1 day.

### Cell viability assay

The cell viability assay was performed as previously described [25]. Briefly, MC3T3-E1 cells in 96-well plates (3×10 ^3^ cells/well) were treated with the corresponding interventions. Osteoblast viability was assessed using 3-(4,5-dimethylthiazol-2-yl)-2,5-diphenyltetrazolium bromide (MTT; MCE, Monmouth Junction, USA). The optical density of the solution was measured at a wavelength of 490 nm with a microplate reader (Bio-Rad, Hercules, USA).

### Analysis of PPARγ protein expression levels in human tissue

PPARγ expression in human bone tissue cells was analysed using published single-cell mRNA sequencing datasets [ 26, 27] after removing the bulk gene background and filtering out cells with fewer than 500 detected transcripts. The matrix of digital gene expression data was transformed with ln(CPM/100+1), and downstream procedures for filtering and reducing dimensionality were performed using Seurat version 4.0.5. Cell type annotation was performed using scMRMA version 1.0. All genes were used for initial principal component analysis, and the first 10 principal components were used for nonlinear dimensionality reduction (t-distributed stochastic neighbourhood embedding, tSNE) analysis.

PPARγ expression in human bone tissue from osteoporotic and healthy individuals was analysed by western blot analysis. A total of 20 osteoporosis patients and 10 healthy controls with T2DM were recruited for this experiment. Bone samples were obtained from patients with fractures during surgery. This study was approved by the Ethics Committee of the First Affiliated Hospital of Fujian Medical University (Fuzhou, China; NO. IEC-FOM-013-2.0).

### Knockdown and overexpression of PPARγ

RNA interference was used to downregulate PPARγ expression. The interfering RNAs (5′-CCTGGCAAAGCATTTGTAT-3′ and 5′-GGGCGATCTTGACAGGAAATT-3′) targeting PPARγ were designed and synthesized by GenePharma Co. (Shanghai, China). In addition, mouse PPARγ DNA was cloned into the GV358 vector by GenePharma Co. The primers for murine PPARγ were as follows: forward, 5′-GAGGATCCCCGGGTACCGGTCGCCACCATGGGTGAAACTCTGGG AGATTC-3′, and reverse, 5′-TCCTTGTAGTCCATACCATACAAGTCCTTGTAGATCTC-3′ . After construction of the PPARγ-RNAi and PPARγ-overexpression vectors, MC3T3-E1 cells were transfected with an infection kit (GenePharma Co.). PPARγ protein expression was confirmed by western blot analysis.

### Animal experiments

Five- to six-week-old male Sprague-Dawley rats were used. After a 1-week acclimation period, the animals were weighed, measured, and divided into two groups: a regular control diet (NC, *n*=20) and a high-fat diet (HFD, *n*=20) group. HFD rats received an HFD for 16 weeks to induce obesity. To induce type 2 diabetes, HFD rats were administered with 30 mg/kg body weight streptozotocin (Sigma Aldrich, St Louis, USA) by injection. Regular control diet rats were injected with an equivalent volume of saline. After the onset of diabetes, a total of 20 model rats were grouped into the metformin-treated group (900 mg/kg/day for 20 weeks, *n*=10) or the T2DM group (gastric perfusion of an equal volume of saline, *n*=10) using a random number table. Body weight and length were recorded once per week for the duration of the study. All rat experiments were approved by the Fujian Medical University Institutional Animal Care and Use Committee.

### Analysis of bone mineral density and bone mineral levels

Bone mineral density was analysed as previously described [ 28, 29] . Briefly, the rats were anaesthetized by an intraperitoneal injection of 10% chloral hydrate (0.03 mL/kg), and bone mineral density and bone mineral levels were measured by dual energy X-ray absorptiometry with a DEXA scanner (GE, Milwaukee, USA).

### ELISA

To analyse serum bone turnover markers, osteocalcin, ALP and TRAP levels in abdominal aorta blood samples were measured. Briefly, after the cells were treated as indicated, the supernatant was collected in a sterile tube. The supernatant was centrifuged at 2850 *g* for 5 min at 4°C and then stored at ‒80°C before analysis. OCN and ALP levels were determined using ELISA kits (Cusabio, Wuhan, China). The functional protein secretion results are expressed as the ratio of ALP and OCN concentrations to the total amount of protein in each group (ALP/PRO, OCN/PRO). The absorbance values were measured at 490 nm using a microplate reader (Thermo Fisher, Waltham, USA) according to the colour reaction.

### Western blot analysis

Control and experimental rats were fasted overnight for 8 h and then anaesthetized by an intraperitoneal injection of 10% chloral hydrate (0.03 mL/kg). Femur tissue was collected, and the muscle and connective tissue were removed, rinsed with saline and stored in liquid nitrogen before analysis. The femur was removed from liquid nitrogen and quickly crushed, and then the bone fragments were ground into powder with liquid nitrogen. The powder was placed in a sterile tube. Then, 1 mL of protein lysate was used per 100 mg of bone tissue, placed on ice for 30 min, shaken once every 10 min, and centrifuged at 12,000 *g* for 10 min at 4°C. The supernatant was stored at ‒80°C. Protein concentrations in femur and MC3T3-E1 cells were determined by using bicinchoninic acid (BCA) protein assay kit (Sigma-Aldrich).

A total of 25 μg of protein was separated by sodium dodecyl sulfate–polyacrylamide gel electrophoresis and transferred to polyvinylidene fluoride membranes (Sigma-Aldrich). The membranes were blocked with 5% nonfat milk in Tris-buffered saline containing 0.1% Tween-20 (TBST) and incubated overnight at 4°C with antibodies against PPARγ (1:200 dilution; Abcam, Cambridge, UK), ATF4 (1:1000 dilution; Abcam), p-AMPK (1:1000 dilution; Abcam), PERK (1:1000 dilution; Abcam), CHOP (1:1000 dilution; Abcam), BMP-2 (1:500 dilution; Abcam) and β-actin (1:100,000 dilution; Abcam). After being washed with TBST three times, the membranes were incubated with horseradish peroxidase-labelled secondary antibodies (1:500 dilution; Abclonal, Wuhan, China) for 1 h at room temperature. Protein signals were measured using an enhanced chemiluminescence detection system (GE). Protein expression levels were normalized to β-actin level.

### Microcomputed tomography (micro-CT)

The first to fourth lumbar vertebrae were collected from the rats and placed in 4% paraformaldehyde for 24 h. The samples were scanned by micro-CT (Scanco Medica, Zurich, Switzerland). The scanning conditions were as follows: 70 kV voltage, 200 μA current, and 9 μm resolution. After the samples were scanned, the images were reconstructed using Mimics 19.0. DataViewer was used to adjust the images.

### Statistical analysis

Statistical analysis was performed using one-way ANOVA. Bivariate correlation analysis was used to analyse the correlation between the levels of pAMPK and the effect of metformin in glucotoxicity osteoblasts. Data are expressed as the mean±SEM. *P* values<0.05 were considered statistically significant.

## Results

### Metformin improves osteoblast differentiation and suppresses PPARγ expression in osteoblasts exposed to glucotoxicity

**Figure 1:**
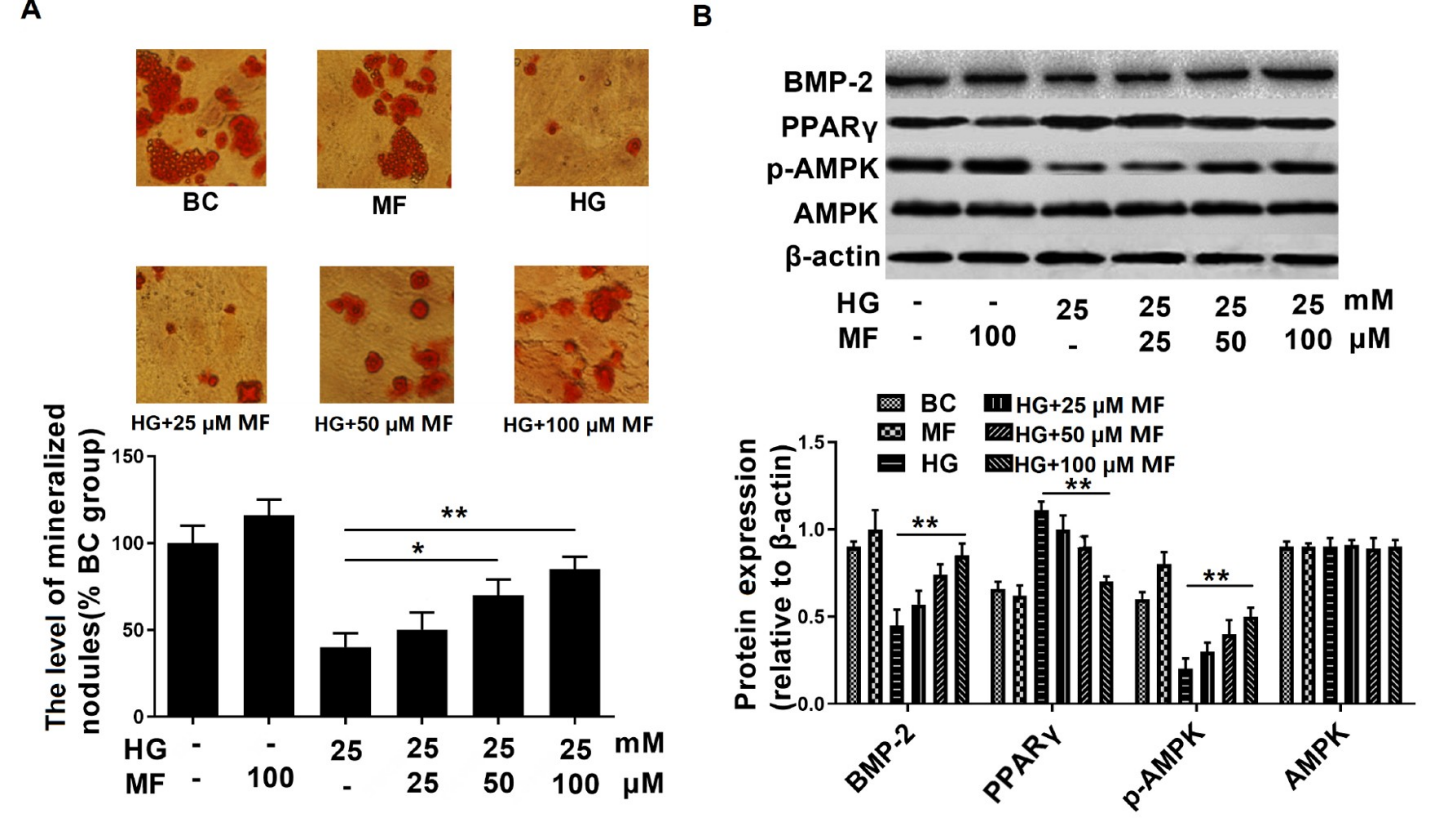
Figure 1 Metformin improves osteoblast differentiation and suppresses PPARγ expression in osteoblasts (A) The osteoblast mineralized nodules were quantitated by Alizarin-Red S staining. (B) The expressions of BMP-2, PPARγ, p-AMPK and AMPK were detected by western blot analysis ( n=3 biologically independent samples). Data are presented as the mean±SEM; * P<0.05, ** P<0.01, two-tailed Student’s t-test.

To examine the effect of metformin on osteoblasts, we treated the streptozotocin-induced osteoporosis cell model with different concentrations of metformin (25, 50, and 100 μM). The results showed that 50 and 100 μM metformin significantly increased mineralization ( Figure 1A) in hyperglycaemic osteoblasts. In the 100 μM metformin treatment group, the protein level of PPARγ was significantly decreased, and the levels of p-AMPK and BMP-2 were increased ( Figure 1B). These findings indicate that metformin improves osteoblast differentiation and suppresses PPARγ expression in osteoblasts.

### PPARγ is highly expressed in the bone tissue of osteoporosis patients

**Figure 2:**
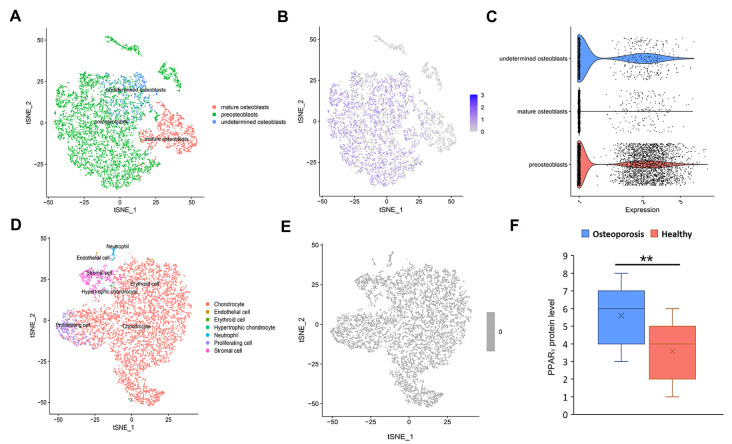
Figure 2 PPARγ is specifically expressed in preosteoblasts of osteoporosis patients Overview of the human cell landscape at the single-cell level for osteoblasts (A) and other cells (D) in bone. PPARγ expression levels in osteoblasts (B,C) and other cells (E) were evaluated using the human cell landscape at the single-cell level. (F) PPARγ protein levels in bone tissue were evaluated by western blot analysis (osteoporosis patients n=20, healthy controls n=10). ** P<0.01, two-tailed Student’s t-test.

To confirm that PPARγ is the specific effector molecule of osteoporosis, we analysed the expression level of PPARγ in human bone tissue using published single-cell mRNA sequencing datasets [ 26, 27] . We found that PPARγ was highly expressed in preosteoblasts (1970/5729 cells) but was rarely expressed in mature osteoblasts (201/1528 cells) ( Figure 2A‒C) and was not expressed in other cells, such as chondrocytes, neutrophils and endothelial cells ( Figure 2D‒E). In addition, we analysed the PPARγ protein levels in the bone tissue of 30 T2DM patients, including 20 osteoporosis patients and 10 healthy individuals ( Figure 2F). The results showed that the PPARγ protein levels in osteoporosis patients were significantly higher than those in healthy controls. Thus, these results suggest that PPARγ is highly expressed in the bone tissue of osteoporosis patients and may play an important role in osteogenic differentiation.

### PPARγ plays an important role in metformin-mediated effect on hyperglycaemic osteoblasts

**Figure 3:**
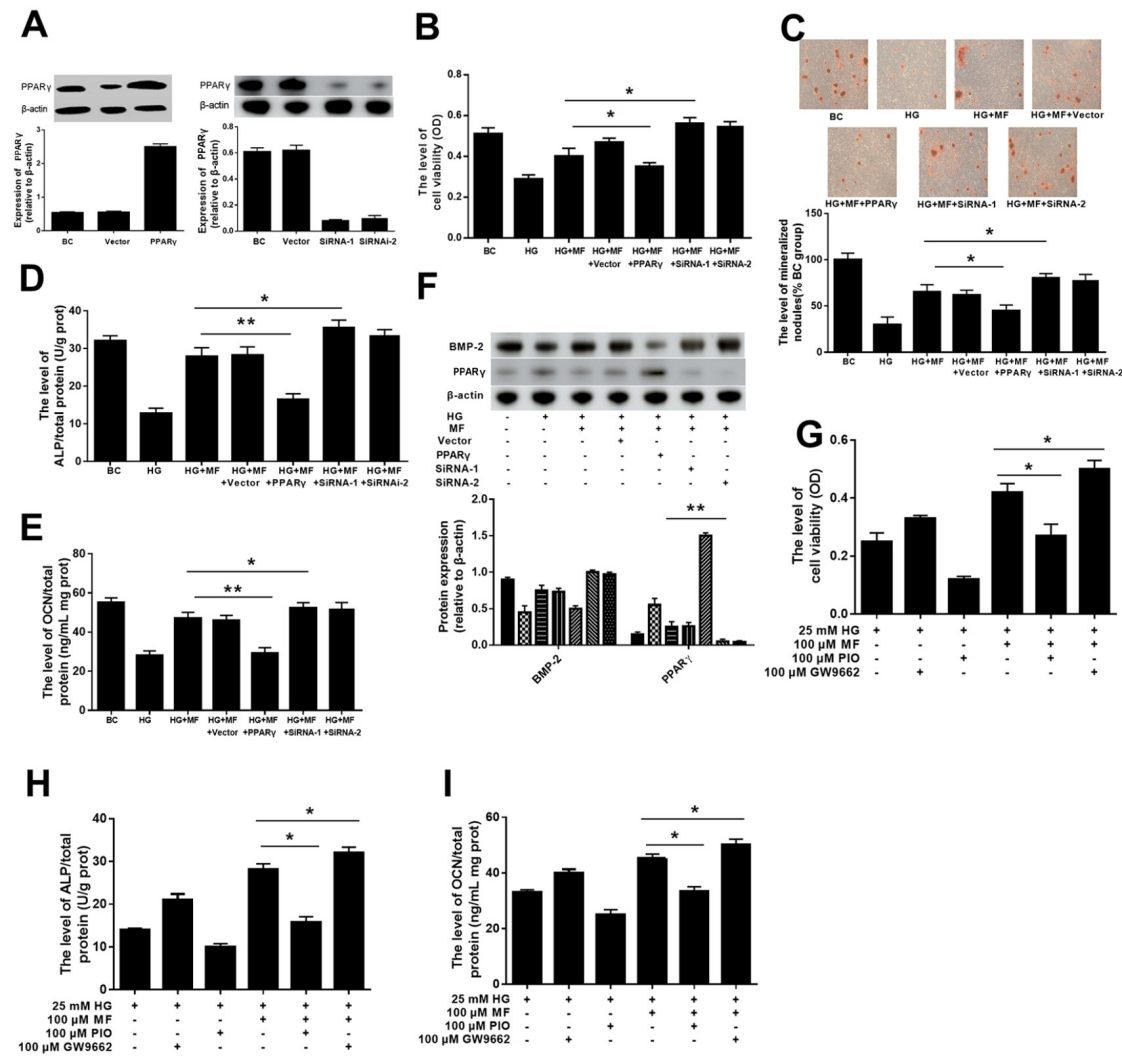
Figure 3 The effects of PPARγ on metformin-attenuating glucotoxicity in osteoblasts (A–F) Relationship between the anti-glucotoxicity effect of metformin and the PPARγ expression. (A) The expression of PPARγ was detected by western blot analysis after silencing or overexpressing PPARγ. (B) The osteoblast viability was assessed by MTT. (C) The osteoblast mineralized nodules were quantitated by Alizarin-Red S staining. (D,E) The influences on the ALP and OCN secretion were measured using ELISA kits. (F) The expression of BMP-2 was detected by western blot analysis in each group. (G–I) Relationship between the anti-glucotoxicity effect of metformin and the PPARγ activity. (G) The osteoblast viability was assessed by MTT. (H,I) The influences on the ALP and OCN secretion were measured using ELISA kits ( n=3 biologically independent samples). Data are presented as the mean±SEM; * P<0.05, ** P<0.01, two-tailed Student’s t-test.

To verify that metformin improves mineralization by affecting PPARγ, we altered the expression or activity of PPARγ in a metformin treatment assay. In this assay, we altered PPARγ levels through overexpression and RNAi-mediated knockdown ( Figure 3A). The results showed that downregulating PPARγ levels improved the metformin-mediated effect on hyperglycaemic osteoblasts; osteoblast viability ( Figure 3B) and mineralization ( Figure 3C), the secretion of ALP ( Figure 3D) and OCN ( Figure 3E), and the expression of BMP2 ( Figure 3F) were increased. Conversely, the increased PPARγ level in osteoblasts weakened the metformin-mediated effect on hyperglycaemic osteoblasts ( Figure 3B‒F).

Furthermore, we explored whether PPARγ activation can alter the effect of metformin on hyperglycaemia-induced osteoporosis. We used a PPARγ inhibitor (GW9662) and agonist (PIO) to selectively block or activate PPARγ activity, respectively. It was found that inhibiting PPARγ activity increased the effects of metformin, increasing osteoblast viability ( Figure 3G), mineralization ( Figure 3H), the secretion of ALP ( Figure 3I) and OCN ( Figure 3J), and the expression of BMP2 ( Figure 3K). In addition, activating PPARγ decreased the effects of metformin ( Figure 3G‒K). These findings indicate that PPARγ plays a key role in the protective effect of metformin on hyperglycaemic osteoblasts.

### PPARγ mediates the effects of metformin through the AMPK pathway

**Figure 4:**
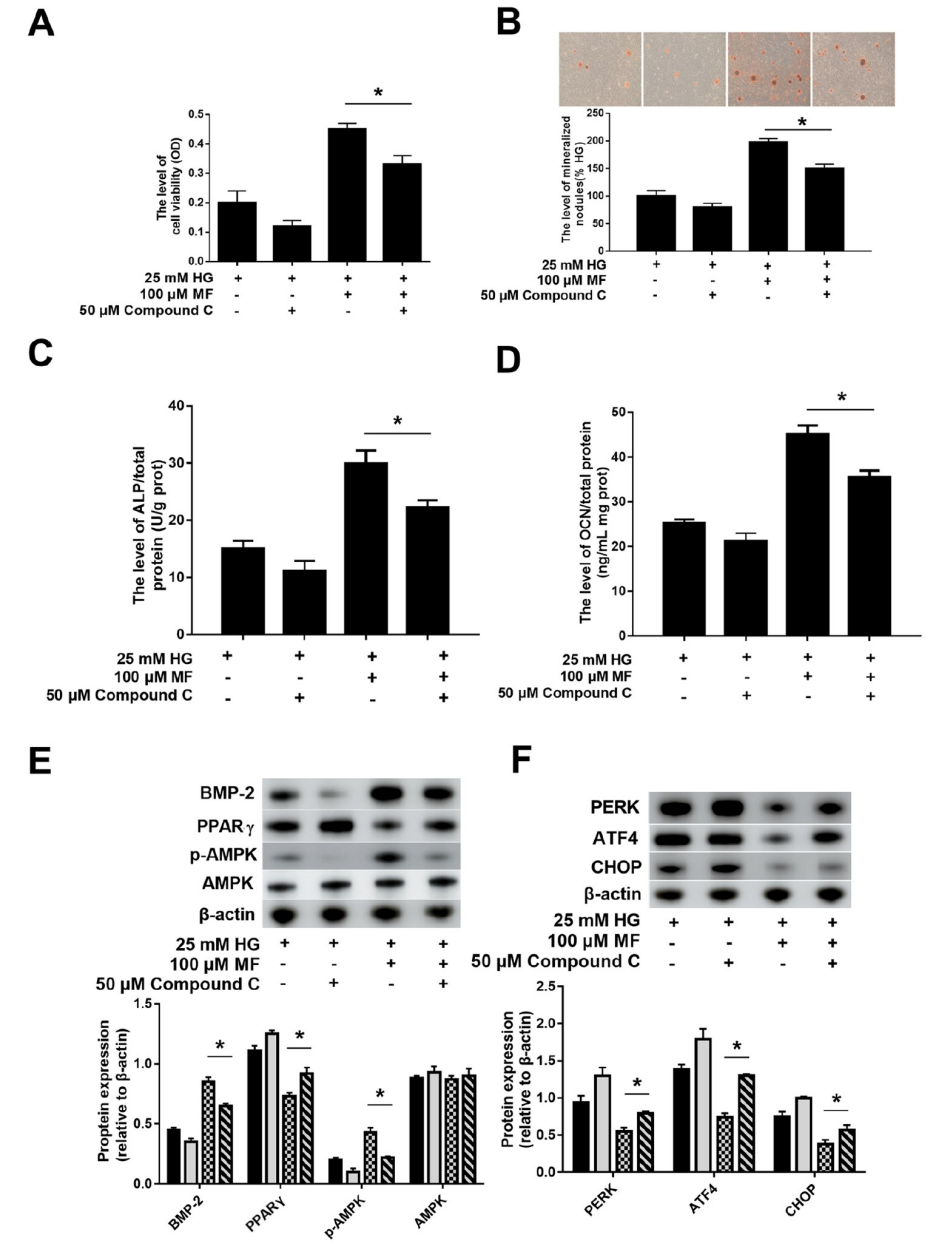
Figure 4 The effect of metformin was weakened with the suppression of AMPK on hyperglycaemic osteoblasts (A) The osteoblast viability was assessed by MTT assay. (B) The osteoblast mineralized nodules were quantitated by Alizarin-Red S staining. The supernatant levels of ALP/total protein (C) and the levels of OCN/total protein (D) in different groups. (E,F) Representative western blots in each group and the ratio of target protein to β-actin ( n=3 biologically independent samples). Data are presented as the mean±SEM; * P<0.05, two-tailed Student’s t-test.

We examined whether PPARγ affects the activity of metformin through the AMPK pathway. Dorsomorphin (compound C) is an AMPK inhibitor. After inhibiting the activity of AMPK, we found that the protective effect of metformin was reduced: compared with those in the HG+MF group, osteoblast viability ( Figure 4A) and mineralization ( Figure 4B), the secretion of ALP ( Figure 4C) and OCN ( Figure 4D), and the expression of BMP2 ( Figure 4E) in the HG+MF+Compound C group were decreased. Compound C also weakened the inhibitory effect of metformin on the expressions of pAMPK, PPARγ, PERK, ATF4, and CHOP ( Figure 4E,F) in hyperglycaemic osteoblasts. These findings suggest that PPARγ affects the activity of metformin through the AMPK pathway.

### PPARγ affects hyperglycaemia-induced osteoporosis through the ERS pathway

**Figure 5:**
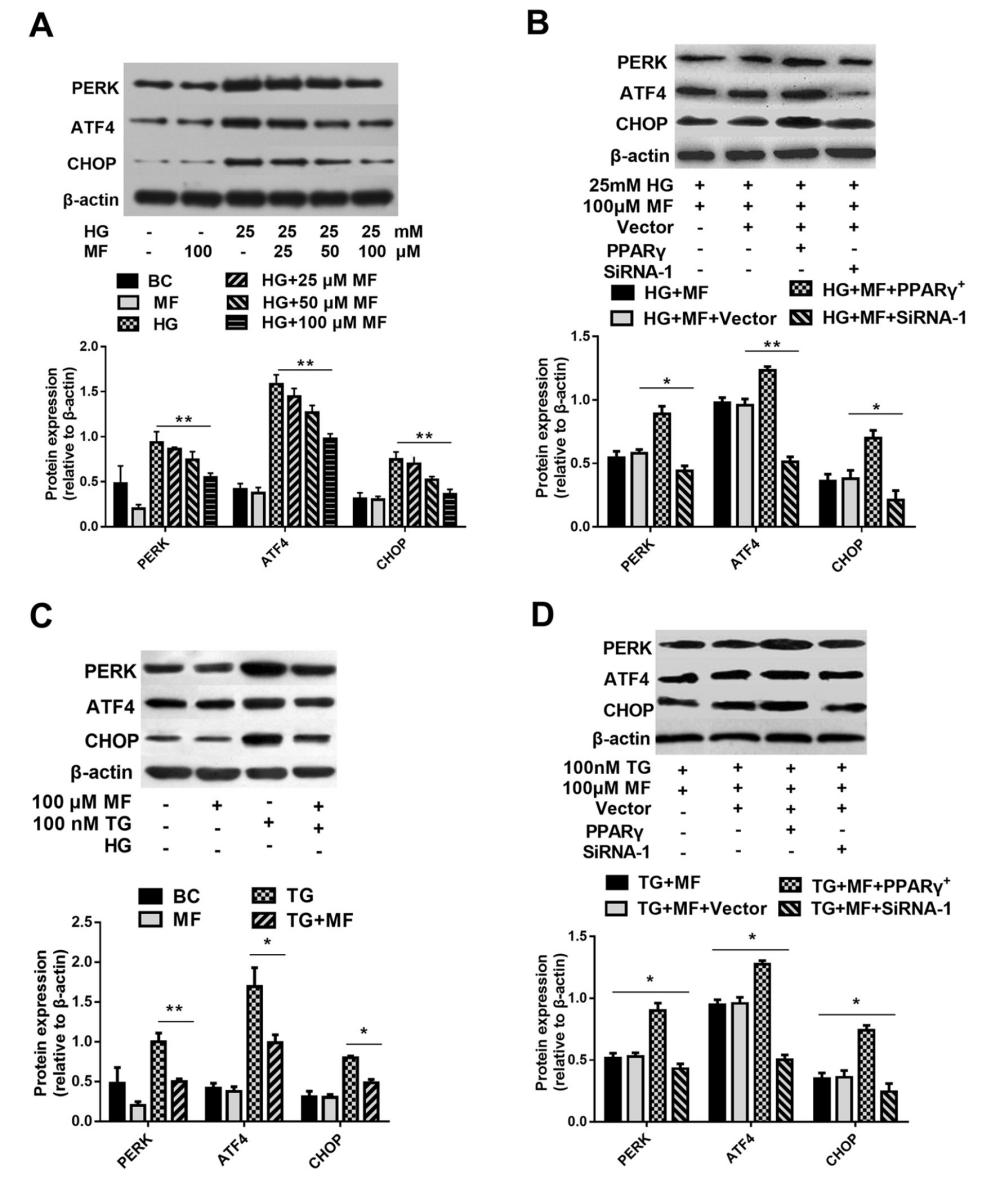
Figure 5 The effect of PPARγ on anti-ERS activity of metformin induced by hyperglycaemic or β-thapsigargin (A) The effect of metformin on the expressions of PERK, ATF4, and CHOP in hyperglycemic osteoblasts. (B) Regulation of PPARγ expression altered the protective effect of metformin on ERS toward hyperglycemic osteoblasts. (C) Metformin inhibited the increased expressions of PERK, ATF4, and CHOP induced by β-thapsigargin. (D) Regulation of PPARγ expression altered the protective effect of metformin on ERS induced by β-thapsigargin in osteoblasts ( n=3 biologically independent samples). Data are presented as the mean±SEM; * P<0.05, ** P<0.01, two-tailed Student’s t-test.

ERS is an important inducer of osteoporosis [20], and metformin inhibits ERS in adipose tissue [21]. Here, we examined the role of PPARγ in ERS after metformin treatment. The results showed that metformin significantly reduced the expression of ERS-associated proteins, such as PERK, ATF4, and CHOP ( Figure 5A), in hyperglycaemic osteoblasts; downregulating PPARγ levels enhanced the effect of metformin on PERK, CHOP and ATF4 in the context of hyperglycemia ( Figure 5B). We also induced PPARγ overexpression in osteoblasts and found that PPARγ overexpression reduced the antagonistic effect of metformin on aberrant gene expression induced by hyperglycaemia in osteoblasts ( Figure 5B).

β-thapsigargin is an activator of ERS and was used to examine whether PPARγ is involved in metformin-mediated alleviation of ERS. Metformin inhibited the increased protein levels of PERK, CHOP and ATF4 induced by β-thapsigargin ( Figure 5C). Silencing of *PPARγ* enhanced the regulatory effect of metformin on the protein levels of PERK, CHOP and ATF4 ( Figure 5D). The protective effect of metformin was weakened by PPARγ overexpression ( Figure 5D). These results suggest that PPARγ is involved in the effects of metformin by alleviating hyperglycaemia-induced ERS.

### PPARγ levels correlate with bone metabolism in metformin treated rats

**Figure 6:**
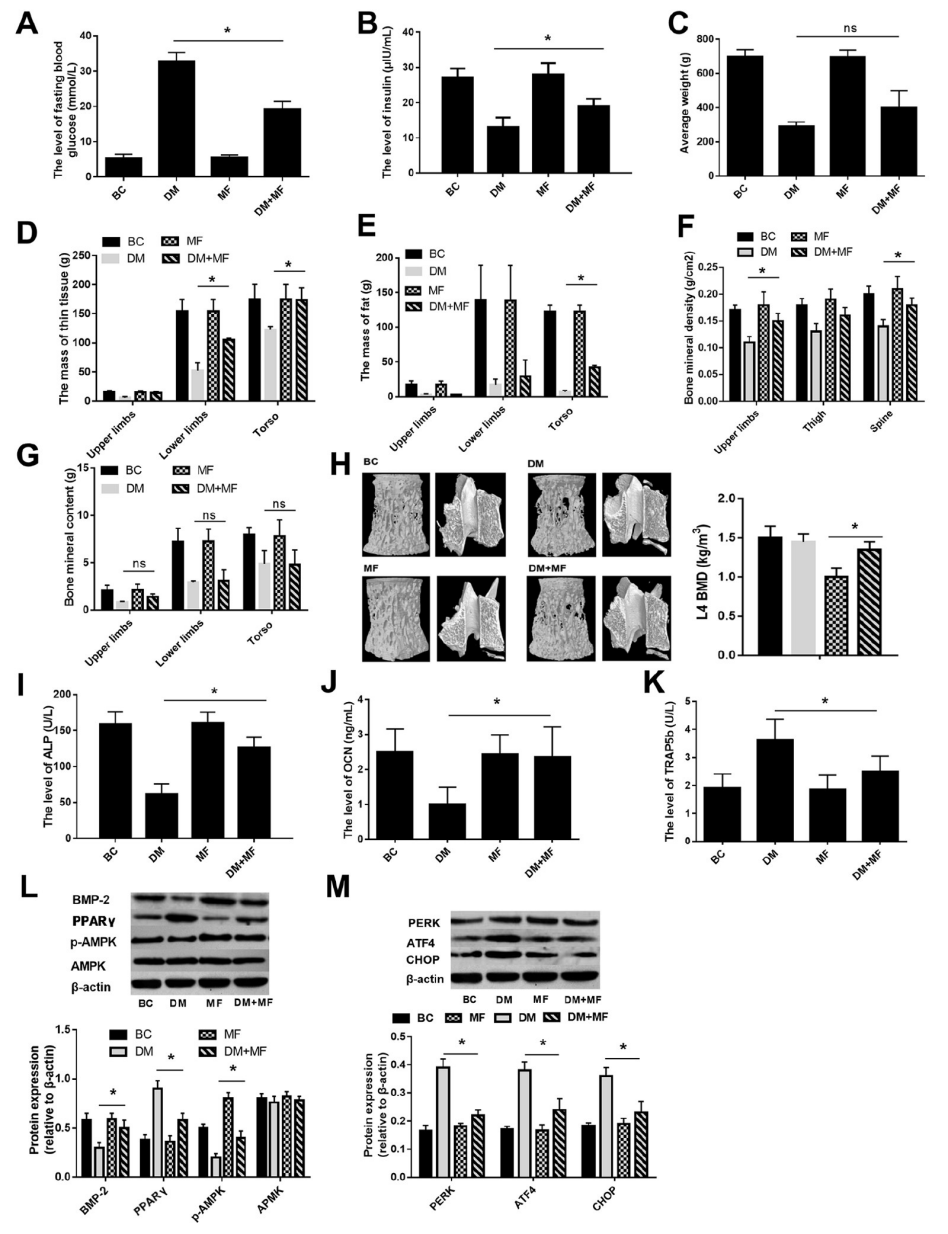
Figure 6 The protective effects of metformin on bone metabolism of diabetic rats The levels of fasting blood glucose (A) and insulin (B) in each group. (C) The average weight of each group. The body compositions (D,E) were detected by DEXA. (F) The bone mineral density was detected by DEXA. (G) The bone mineral content was detected by DEXA. (H) The micro-CT showed the bone quantity in torso tissue. (I–K) The bone turnover markers were measured using ELISA kits. (L,M) Representative western blots in each group and the ratio of target protein to β-actin (NC, n=20; HFD, n=20). Data are presented as the mean±SEM; * P<0.05, two-tailed Student’s t-test.

To examine the protective effect of metformin against hyperglycemia-induced osteoporosis, the bone metabolism of diabetic rats was examined after treatment with metformin. Diabetes was induced in rats by a high-fat diet and streptozotocin. The results showed that metformin reduced fasting blood glucose ( Figure 6A) and increased insulin secretion ( Figure 6B), the abundance of thin tissue ( Figure 6D), the fat mass in the torso ( Figure 6E), and BMD in the whole body ( Figure 6F). However, no significant difference was observed in the weight ( Figure 6C) or bone mineral levels ( Figure 6G) between the DM and DM+MF groups. The micro-CT results showed that metformin treatment promoted bone formation in torso tissue ( Figure 6H). Bone metabolism markers were examined, and metformin treatment increased the levels of ALP ( Figure 6I) and OCN ( Figure 6J) and decreased the secretion of TRAP5b ( Figure 6K). These results demonstrate that metformin improves hyperglycaemia-induced osteoporosis.

Furthermore, we analysed the PPARγ pathway in the DM and DM+MF groups. The results showed that metformin treatment increased the expressions of BMP2 and pAMPK ( Figure 6L) and reduced the expressions of PPARγ ( Figure 6L), PERK, ATF4, and CHOP ( Figure 6M) in femur tissues. These findings indicate that metformin improves bone metabolism in diabetic rats, which correlates with the PPARγ-related pathway.

## Discussion

In this study, we demonstrated that metformin enhanced osteoblast differentiation and downregulated the protein level of PPARγ in an osteoporosis cell model. Increasing the activity of PPARγ weakened these effects of metformin, whereas inhibiting the activity of PPARγ had the opposite effect. Additionally, we found that metformin regulated PPARγ function through the AMPK pathway. PPARγ mediates the effect of metformin via the ERS pathway. Furthermore, we found a relationship between the effect of metformin on bone metabolism and the activity of PPARγ in diabetic rats.

Previous experiments confirmed that the pathway by which metformin protects against hyperglycaemia in osteoblasts is related to a reduction in PPARγ [30]. However, what is the association between metformin and PPARγ? There is no related literature available. It has been verified that metformin protects against hyperglycaemia by regulating AMPK signalling [31]. Furthermore, our results showed that AMPK activity is negatively correlated with PPARγ expression level. Therefore, we hypothesized that metformin-mediated regulation of PPARγ expression might be related to AMPK. Thus, we inhibited AMPK and then observed whether the effect of metformin could be diminished in hyperglycaemic osteoblasts and whether metformin still could affect the expression of PPARγ. Our results revealed that the effect of metformin in this context was, to a certain extent, AMPK dependent.

During diabetes, bone strength is decreased, and bone fragility is increased, which is induced by high glucose toxicity, leading to the occurrence and development of diabetic osteoporosis [32]. Our results indicate that metformin decreases the expressions of ERS-related proteins in hyperglycaemic osteoblasts. Current research also suggests that metformin has a protective effect on hyperglycaemic osteoblasts [33]. We confirmed that metformin could inhibit PPARγ expression and thus attenuate glucotoxicity in osteoblasts. Next, we examined the ways by which metformin mediates its effects. Previous research has shown that ERS is a central mechanism of injury induced by high glucose in osteoblasts [15]. Studies from other laboratories have also demonstrated that metformin can prevent renal fibrosis by relieving ERS [34]. Our study verified the efficacy of metformin on ERS in osteoblasts.

In summary, we show that metformin enhances osteoblast differentiation by regulating PPARγ expression. Furthermore, the AMPK and ERS pathways are upstream and downstream of PPARγ, respectively. We conclude that: (i) PPARγ is the effector through which metformin prevents osteoporosis. (ii) PPARγ levels are related to osteoporosis in diabetes patients. (iii) Metformin prevents hyperglycaemia-induced osteoporosis by regulating the AMPK-PPARγ-ERS axis. Our findings will expand treatment strategies for hyperglycaemia-induced osteoporosis and may also expand metformin treatment to other PPARγ-related diseases.
